# Efficacy and safety of small molecule drugs in the treatment of pityriasis rubra pilaris—A systematic review

**DOI:** 10.3389/fmed.2025.1544197

**Published:** 2025-02-25

**Authors:** Xiaofang Zhang, Kebo Wei, Hongxia Song, Xi Chen, Jiao Yang, Jianmei Zhao, Yugu Jiang, Xin He

**Affiliations:** ^1^Department of Dermatology, Sichuan Mianyang 404 Hospital, Mianyang, Sichuan, China; ^2^Department of Dermatology, Hospital of Chengdu University of TCM, Chengdu, Sichuan, China; ^3^Department of Dermatology, Chengdu Integrated TCM and Western Medicine Hospital, Chengdu, Sichuan, China

**Keywords:** pityriasis rubra pilaris, systematic review, small molecule drugs, efficacy and safet, Janus kinase (JAK) inhibitors, PDE-4 inhibitor

## Abstract

**Background:**

Pityriasis rubra pilaris is a chronic, scaly, keratotic skin disease, mainly manifested as scaly plaques and keratinized hair follicles. This condition significantly impacts the patient’s quality of life and is considered one of the intractable diseases in dermatology. Currently, no satisfactory clinical treatment options are available for this condition, presenting a considerable challenge for dermatologists. We conducted this systematic evaluation to assess the therapeutic potential of existing small molecule drugs for this disease.

**Objectives:**

To conduct a systematic review of the existing literature on the use of small molecule drugs for treating pityriasis rubra pilaris and to evaluate their clinical effectiveness and safety.

**Methods:**

We conducted a systematic review of all the literature on small molecule drugs for the treatment of Pityriasis rubra pilaris and searched several databases until November 2024, including PubMed, Embase, Web of Science, and the Cochrane Library.

**Results:**

A total of 16 patients with pityriasis rubra pilaris from 11 publications were included. The small molecule drugs, including apremilast, upadacitinib, abrocitinib, and tofacitinib, demonstrate good efficacy and safety in the treatment of pityriasis rubra pilaris across all ages, particularly in patients who have failed systemic therapy and have a poor response to biological agents. However, the conclusions are limited by the small sample size and need to be further confirmed through large-scale randomized controlled clinical trials.

**Conclusion:**

Small molecule drugs demonstrate favorable clinical efficacy and safety in the treatment of refractory pityriasis rubra pilaris, exhibiting a relatively rapid onset and a high safety profile. However, the findings in the literature may be affected by publication bias.

## Introduction

Pityriasis rubra pilaris (PRP) is a rare, chronic, scaly, keratotic inflammatory skin disease characterized by scaly plaques and keratotic follicular papules. Most patients are often accompanied by palmoplantar keratoderma. Histologically, an alternating pattern of orthokeratosis and parakeratosis is considered the hallmark of PRP (1). The disease can be categorized into two types: familial and acquired. The familial type is more prevalent in children, while the acquired type is more common in adults (2). According to the various manifestations observed in patients, the condition is primarily classified into five types: type I (classical adult), type II (atypical adult), type III (classical juvenile), type IV (circumscribed juvenile), and type V (atypical juvenile). Type I (classical adult) is the most common clinical type, with the most typical symptoms and a better prognosis, with most patients resolving spontaneously. Type II (atypical adult) is less common clinically, with atypical symptoms and eczema-like changes. Type III (classical juvenile) is usually found in children aged 5–10 years, with clinical manifestations similar to those of type I (classical adult), and some patients have a history of acute infections, with a high rate of spontaneous remission. Type IV (circumscribed juvenile) is rare clinically, and the rash is mostly confined to the elbow, Type V (atypical juvenile) develops shortly after birth and presents with erythema, hyperkeratosis, and follicular keratosis, often with a family history, and rarely resolves spontaneously. Additionally, some studies have reported the presence of pityriasis rubra pilaris associated with HIV infection (VI PRP) (3). This type VI PRP may be associated with concurrent acne conglobata, hidradenitis suppurativa, or lichen spinulosus—conditions that fall under the umbrella of the follicular occlusion tetrad. Patients with type VI frequently experience erythroderma (3–6). Recently, two new entities that are clinically and histologically similar to PRP have been identified: CARD14-associated papulosquamous eruption (CAPE) and facial discoid dermatitis (FDD), as well as paraneoplastic PRP (7–9). SARS-CoV-2 infection-associated and post-vaccination erythematous furuncle rash has also been reported in the literature (10, 11). Four cases of childhood PRP rash due to acute infection have been reported in the literature, with spontaneous resolution of the lesions within 3 months without recurrence (12). PRP can affect individuals of all ages, with slight increases in prevalence observed during early childhood and among those aged 50–60 years (13). It is unclear whether PRP has a predilection for men or women (14, 15).

The exact etiology and pathogenesis of PRP remain unclear and may be associated with factors such as heredity, endocrine dysfunction, vitamin A deficiency, autoimmune diseases, infections, trauma, and vaccination, among others (16–20). A number of foreign studies indicate that this disease significantly impacts the quality of life of patients, imposing a considerable psychological burden. This burden can lead to depression, anxiety, and even suicidal tendencies, ultimately affecting patients’ ability to perform daily activities (21). Current treatment approaches primarily rely on clinical experience and case reports, encompassing systemic regimens such as acitretin, methotrexate, glucocorticoids, and cyclosporine, among others (22, 23). However, these treatments often fail to yield satisfactory results. As our understanding of this disease deepens, biological agents have emerged as viable treatment options, including secukinumab and ixekizumab (24–26). Although biological agents have yielded some positive outcomes, there are still patients who experience poor efficacy and may even suffer from serious adverse reactions following treatment (27, 28). For certain refractory patients, small molecular drugs appear to demonstrate improved safety and efficacy. This article systematically evaluates the effectiveness and safety of small molecular drugs in the treatment of this disease.

## Materials and methods

### Search strategy

The systematic review was conducted and reported by the Preferred Reporting Items for Systematic Reviews and Meta-Analyses (PRISMA) statement (29). We searched several databases, including PubMed, Embase, Web of Science, and the Cochrane Library, until November 2024. In PubMed, we used the following keywords: “pityriasis rubra pilaris,” “Janus kinase (JAK) inhibitors,” “small-molecule drugs,” “apremilast,” “upadacitinib,” “abrocitinib,” and “tofacitinib.” After retrieving the relevant literature, the two authors screened the results by reading the titles and abstracts. They then reviewed the full texts of the screened literature, and after further filtering, the final selection of literature was included.

### Eligibility criteria

We included all studies, such as randomized controlled trials (RCTs), retrospective studies, and case reports, that focused on the treatment of PRP with small-molecule drugs, including apremilast, upadacitinib, abrocitinib, and tofacitinib. Only English articles were considered.

### Study selection and data extraction

After retrieving the relevant literature, the two authors screen the articles by reviewing the titles and abstracts. They then read the full texts of the screened literature, and after further filtering, the final selection of literature is included.

### Statistical analysis

Due to the limited availability of literature and case studies, we have included basic information, efficacy, and safety data for all patients in the article in tabular form. This table allows for a more intuitive assessment of patient’s efficacy and safety.

## Results

**FIGURE 1:**
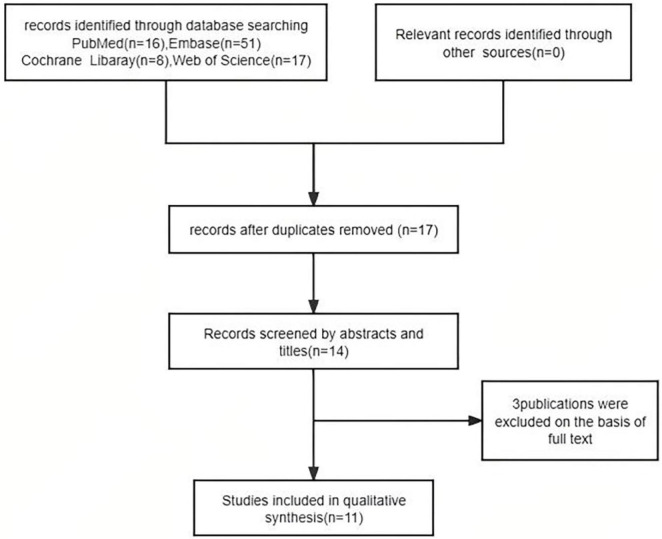
Selection of the included studies.

We reviewed 92 papers and ultimately included 11 in the final study (30–40), which comprised 3 Jak inhibitors (Abrocitinib Upadacitinib Tofacitinib) and Phosphodiesterase-4 Inhibitors(Apremilast) (Figure 1).

### JAK inhibitors

The literature reports three types of JAK inhibitors for the treatment of PRP (Table 1). Among them, Abrocitinib and Upadacitinib are highly selective JAK 1 inhibitors. Tofacitinib is a generation of JAK inhibitors, which can effectively inhibit the activity of JAK1 and JAK3, and block the signal transduction of multiple inflammatory cytokines. Among the 12 patients studied, nine were women. The ages of the patients ranged from 13 to 81 years, and the duration of the condition varied from 2 months to 26 years. In terms of dose and frequency, among patients taking Abrocitinib, all patients were on 100 mg once daily (30), and among patients taking Upadacitinib, all patients were on 15 mg once daily, except for one patient whose dose was adjusted to 30 mg once daily after 2 weeks of administration (38). The basal dose of Tofacitinib was 5 mg bid in all patients and was reduced to 5 mg once daily after 1 month in one patient (38). In previous systemic treatments, six patients were treated with acitretin, seven with biological agents, one with the small molecule drug apremilast, one with methotrexate, one with systemic hormone therapy, one with phototherapy, and one with antihistamines. In terms of efficacy, all 12 patients (100%) experienced complete alleviation of symptoms within 6 months, and six patients (50%) achieved complete symptom relief in approximately 3 months. Regarding safety, two patients reported adverse reactions, specifically acne, and headache, which resolved spontaneously. These findings indicate that JAK inhibitors demonstrate both effective therapeutic outcomes and a favorable safety profile for the treatment of PRP.

**TABLE 1:** Characteristics of the included studies.

References	Age/sex	Type	Dose regimen	Concomitant treatments	Disease course of PRP (Year)	follow-up time (months)	Response	Flares	Previous treatment	Adverse events
**Abrocitinib**										
Li et al. (30)	Female (27)	N/A	100 mg once daily	Topical glucocorticoids moisturizer	0.5	3 months	Completely alleviated	N/A	Secukinumab Ixekizumab	N/A
	Female (78)	N/A	100 mg once daily	Topical glucocorticoids moisturizer	1	3 months	Completely alleviated	N/A	N/A	N/A
	Female (30)	N/A	100 mg once daily	Topical glucocorticoids moisturizer	3	3 months	Completely alleviated	N/A	Acitretin	N/A
	Male (35)	N/A	100 mg once daily	Topical glucocorticoids moisturizer	11	3 months	Completely alleviated	N/A	Apremilast	N/A
	Male (68)	N/A	100 mg once daily	Topical glucocorticoids moisturizer	0.5	3 months	Completely alleviated	N/A	N/A	N/A
**Upadacitinib**										
Li et al. (35)	Man (42)	N/A	15 mg once-daily	N/A	20 years	6 months	completely alleviated	N/A	Acitretin Secukinumab	N/A
	Female (13)	N/A	15 mg once-daily	N/A	5 years	6 months	completely alleviated	N/A	Secukinumab	Acne
Xiaoyuan et al. (36)	Female (13)	N/A	15 mg once-daily	N/A	1 year	1 month	completely alleviated	N/A	Acitretin Methotrexate Secukinumab	N/A
Song et al. (37)	Female (81)	N/A	15 mg once-daily	N/A	1 year	4 months	completely alleviated	N/A	Dupilumab Systemic corticosteroids Acitretin Ixekizumab	N/A
Saad et al. (38)	Female (26)	Type III	15 mg/day 2 weeks 30 mg/day	N/A	24 years	2 months	completely alleviated	N/A	Isotretinoin, Phototherapy Ustekinumab Ixekizumab	Headache
**Tofacitinib**										
Tan et al. (39)	Female (39)	N/A	5 mg bid 1 month 5 mg qd 1 month	N/A	20 days	2 months	completely alleviated	N/A	Acitretin Olopatadine	N/A
Ying et al. (40)	Female (57)	Type 1	5 mg bid	N/A	0.2 year	3 months	completely alleviated	N/A	Acitretin Ixekizumab	N/A
**Apremilast**										
Krase et al. (31)	Male (70)	N/A	10 mg/d to 30 mg twice daily	N/A	0.8	8 months	6 months completely alleviated	N/A	Acitretin Methotrexate Prednisone Cyclosporine Infliximab	Fmild fastrointestinal up-set
Molina-Figuera et al. (32)	Female (61)	Type 1	30 mg twice daily	N/A	0.3	7 months	3 months completely alleviated	N/A	Oral corticosteroid Acitretin Methotrexate Adalimumab Cyclosporine	N/A
Pellonnet et al. (33)	Male (47)	N/A	10 mg/d to 30 mg twice daily	N/A	0.8	7 months	2 month completely alleviated	N/A	Ointments Acitretin UVB	N/A
Cho et al. 2018 (34)	Female (60)	Type 1	30 mg/day	N/A	4	6 months	2 months significant improvements	N/A	Steroid ointments Oral retinoic Acid Vitamin D3 Analog Salicylic acid	Mild headaches

PRP, pityriasis rubra pilaris; UVB, ultraviolet radiation b.

### Apremilast

A total of four patients were reported to have taken apremilast to treat PRP (Table 1). The patients’ ages ranged from 47 to 70 years, and the duration of the disease varied from three months to 4 years. The frequency and dosage of the drug varied among the four patients, with two patients starting at 10 mg/day and increasing to the recommended maintenance dose of 30 mg twice a day after 5 days (31, 33). The other two patients were 30 mg once daily (32) and 30 mg twice daily (34), respectively.

Following treatment with apremilast, nearly all patients experienced complete alleviation of their symptoms. One patient had complete symptom relief after 2 months, while two other patients achieved complete relief after three months and 6 months, respectively. One patient’s symptoms showed significant improvement after 2 months of treatment, and the relief continued over the subsequent 6 months. Regarding safety, two patients (50%) reported mild adverse reactions, including gastrointestinal discomfort and mild headaches, which resolved naturally. Consequently, apremilast demonstrates a positive effect on the treatment of PRP. However, further clinical studies with large, multicenter samples are necessary to validate these findings.

## Discussion

Pityriasis rubra pilarisrefers to a rare chronic inflammatory skin disease characterized by yellow-red scales and keratinized hair follicle papules (41). The quality of life of patients is significantly affected (42). The current treatment primarily involves the use of immunosuppressants; however, the effects vary among patients, and some experience limited therapeutic benefits (23). With the continued deepening of our understanding of this disease, research has identified the IL-23/T-helper cell (Th17) axis as a major contributor to the pathogenesis of PRP (19). IL-23 is mainly produced by dendritic cells and macrophages, and up-regulation of the IL-23 receptor by certain SNP alleles may enhance STAT signaling, which promotes the differentiation of helper T cell 17 (Th17) and contributes to keratinocyte activation and hyperproliferation (43). Therefore, a variety of biological agents are utilized for treatment; however, some patients continue to experience inadequate outcomes (44). At the same time, some literature has reported that the symptoms associated with various biological agents have not improved significantly (40). Cytokines play a key role in many biological responses and shape the immune response. When cytokines are produced or their biological activity is faulty, the homeostatic balance of the immune response is altered, leading to the development of a variety of conditions such as autoimmune and inflammatory diseases (45). However, cytokine binding to receptors initiates corresponding inflammatory signaling, and receptor signaling is dependent on JAKs. Therefore, inhibition or blockade of the JAK-STAT signaling pathway has become an important direction for targeted therapy of immune-mediated inflammatory diseases. Phosphodiesterase 4 (PDE4) inhibitors can downregulate the production of various pro-inflammatory cytokines, such as IL-23, and are widely used in various immune-inflammatory diseases (46). We reported on a total of 16 patients by searching the literature, of whom 9 (56%) used biological agents, and clinical symptoms were challenging to control. After administering small molecule drugs, the symptoms were significantly alleviated. The ages of the patients ranged from 13 to 81 years, encompassing both teenagers and the elderly, all of whom achieved favorable therapeutic outcomes. In terms of safety, 4 patients reported common adverse reactions to the medication, which resolved spontaneously, indicating that small molecule drugs demonstrate both efficacy and safety in the treatment of PRP. Compared to apremilast, jak inhibitors have shown better efficacy, with most patients achieving complete remission, as well as better efficacy in patients who have failed apremilast therapy. Considering that the clinical manifestations and prognosis of different types of PRP vary greatly, and that type I (classical adult) and type III (classical juvenile) have a high rate of self-healing, some acute PRP should be actively screened for the cause of the disease to avoid over-treatment. Currently, treatment options for PRP are limited. Though the findings in the literature may be affected by publication bias, small molecule drugs appear to be a viable treatment alternative, particularly for patients who have not responded to multiple drug therapies. However, due to factors such as the small sample size and the low quality of existing literature, further clinical research is necessary to validate the clinical efficacy of small molecule drugs.

## Conclusion

This study reported on a total of 16 patients, of whom 9 (56%) used biological agents, and clinical symptoms were challenging to control. After administering small molecule drugs, the symptoms were significantly alleviated. The ages of the patients ranged from 13 to 81 years, encompassing both teenagers and the elderly, all of whom achieved favorable therapeutic outcomes. In terms of safety, four patients reported common adverse reactions to the medication, which resolved spontaneously, indicating that small molecule drugs demonstrate both efficacy and safety in the treatment of PRP. Currently, treatment options for PRP are limited. Small molecule drugs appear to be a viable treatment alternative, particularly for patients who have not responded to multiple drug therapies. However, due to factors such as the small sample size and the low quality of existing literature, further clinical research is necessary to validate the clinical efficacy of small molecule drugs.

## Data Availability

The original contributions presented in the study are included in the article/supplementary material, further inquiries can be directed to the corresponding author.
